# Endothelial RhoA GTPase is essential for *in vitro* endothelial functions but dispensable for physiological *in vivo* angiogenesis

**DOI:** 10.1038/s41598-019-48053-z

**Published:** 2019-08-12

**Authors:** Fatema Tuz Zahra, Md Sanaullah Sajib, Yusuke Ichiyama, Racheal Grace Akwii, Paul E. Tullar, Christopher Cobos, Shelby A. Minchew, Colleen L. Doçi, Yi Zheng, Yoshiaki Kubota, J. Silvio Gutkind, Constantinos M. Mikelis

**Affiliations:** 1 Department of Pharmaceutical Sciences, School of Pharmacy, Texas Tech University Health Sciences Center, Amarillo, Texas 79106 USA; 2 Department of Anatomy, Keio University School of Medicine, 35 Shinanomachi, Shinjuku-ku, Tokyo, 160-8582 Japan; 3 Department of Ophthalmology, Shiga University of Medical Science, Seta Tsukinowa-cho, Otsu, Shiga 520-2192 Japan; 4 Department of Obstetrics and Gynecology, School of Medicine, Texas Tech University Health Sciences Center, Amarillo, Texas 79106 USA; 5 College of Arts and Sciences, Marian University Indianapolis, Indianapolis, Indiana 46222 USA; 6 Cancer and Blood Diseases Institute, Cincinnati Children’s Hospital Medical Center, University of Cincinnati College of Medicine, Cincinnati, Ohio 45229 USA; 7 Department of Pharmacology, UCSD, San Diego, California 92093 USA

**Keywords:** Angiogenesis, RHO signalling

## Abstract

Imbalanced angiogenesis is a characteristic of several diseases. Rho GTPases regulate multiple cellular processes, such as cytoskeletal rearrangement, cell movement, microtubule dynamics, signal transduction and gene expression. Among the Rho GTPases, RhoA, Rac1 and Cdc42 are best characterized. The role of endothelial Rac1 and Cdc42 in embryonic development and retinal angiogenesis has been studied, however the role of endothelial RhoA is yet to be explored. Here, we aimed to identify the role of endothelial RhoA in endothelial cell functions, in embryonic and retinal development and explored compensatory mechanisms. *In vitro*, RhoA is involved in cell proliferation, migration and tube formation, triggered by the angiogenesis inducers Vascular Endothelial Growth Factor (VEGF) and Sphingosine-1 Phosphate (S1P). *In vivo*, through constitutive and inducible endothelial RhoA deficiency we tested the role of endothelial RhoA in embryonic development and retinal angiogenesis. Constitutive endothelial RhoA deficiency, although decreased survival, was not detrimental for embryonic development, while inducible endothelial RhoA deficiency presented only mild deficiencies in the retina. The redundant role of RhoA *in vivo* can be attributed to potential differences in the signaling cues regulating angiogenesis in physiological versus pathological conditions and to the alternative compensatory mechanisms that may be present in the *in vivo* setting.

## Introduction

The Rho family of GTPases is part of the Ras superfamily, which comprises over 150 members in human, with evolutionarily conserved orthologs found in yeast and plants^1,2^. Rho GTPases are present in all eukaryotes, and play important role in the regulation of actin and microtubule cytoskeleton, cell migration and invasion, cell polarity, vesicle trafficking, regulation of gene expression and cell cycle progression^1,3^. The 20 known members of the Rho GTPase family are characterized as typical and atypical, based on whether their regulation depends on the interaction with the Rho-specific guanine nucleotide exchange factors (GEFs) and the GTPase-activating proteins (GAPs) for activation and inactivation respectively^3^. The best-known members of the typical GTPase family are Ras homolog gene family, member A (RhoA), Ras-related C3 botulinum toxin substrate 1 (Rac1) and cell division control protein 42 (Cdc42), which are known regulators of the actin cytoskeleton and whose timely and spatially coordinated activation leads to cell migration and morphology^3,4^. Typically, RhoA regulates stress fiber formation and Rac1 and Cdc42 control lamellipodia and filopodia formation respectively^5^, whereas more recent findings highlight their role in less common cytoskeletal structures, such as podosome and invadopodia formation in cancer cells^6^. Rho GTPase activation is stimulated through activation of a variety of cell surface receptors, including G protein-coupled receptors (GPCRs) such as lysophosphatidic acid (LPA) and bombesin receptors, tyrosine kinase growth factor receptors such as platelet-derived growth factor (PDGF) and epidermal growth factor (EGF) receptors, as well as cell adhesion molecules such as integrins, cadherins and immunoglobulin superfamily members^7^.

Angiogenesis is a well-coordinated process, important for the vascular development in all higher organisms, and its deregulation initiates or augments the development of many pathological conditions^8^. Small GTPases are actively involved in many steps of the angiogenic process, as the distinct role of RhoA, Rac1 and Cdc42 and others has been reported for endothelial cell migration, proliferation, basement membrane degradation, morphogenesis, capillary survival and barrier function^9^. Furthermore, they have all been demonstrated as downstream effectors of potent angiogenesis inducers, such as vascular endothelial growth factor (VEGF)^10^ and sphingosine-1-phosphate (S1P)^11,12^. Global deficiency of each Rho GTPase leads to embryonic lethality, indicative of their significance on embryonic development: *Cdc42*-knockout mice die prior to embryonic day 7.5 (E7.5)^13^, *Rac1*-knockout mice die prior to E9.5 due to germ-layer formation deficiencies^14^, whereas viable *RhoA-*knockout mice have not been reported^15^. The development of conditional deficient mice provides a better tool to evaluate the significance and function of the protein of interest on a specific tissue. Endothelial-specific deficiency of Cdc42 (through the Tie2-Cre promoter) led to embryonic lethality by E9-10 due to deficiencies in lumen formation and failed blood circulation. Inducible endothelial Cdc42 deficiency during mid-gestation (through the Cad5-Cre^ERT2^ promoter), also led to embryonic lethality, demonstrating the importance of Cdc42 during vessel formation and endothelial cell polarity during angiogenesis^16^. In the same mouse model, it was also shown that *Cdc42* deletion affects retinal angiogenesis^16,17^. Rac1 endothelial-specific deficiency also led to embryonic lethality during midgestation (E9.5) due to defective development of major vessels and complete lack of small vessel branching^18^. Inducible homozygous endothelial deletion of *Rac1* at E10 led to remarkable hemorrhage at E15.5 and to the reduction of the vascular area and number of branching points in the embryonic back skin, while inducible deficiency in the developing retina resulted in reduction of vascular area and branching points at P8^19^. Despite the detailed knowledge on the role of Cdc42 and Rac1 on developmental angiogenesis, the precise role of RhoA has yet to be elucidated.

We previously showed that combined global deficiency of two known RhoA GEFs, PDZ-RhoGEF and leukemia-associated Rho GEF (LARG) blocked RhoA activation downstream of the Gα_12/13_ GPCRs, leading to embryonic lethality during midgestation due to branching deficiencies of the cranial vessels and in the embryonic vascular network in the placenta^20^. The above prompted us to explore the biological role of endothelial RhoA during embryonic development and retinal angiogenesis. We engineered constitutive and inducible endothelial-specific RhoA deficiency through the *Tie2-Cre* and *Cdh5-Cre*^*ERT2*^ promoters respectively, studied the impact of endothelial *RhoA* deletion in embryonic survival and retinal angiogenesis, and compared the *in vivo* data with the *in vitro* outcome of RhoA deficiency in endothelial cell functions, under stimulation by potent angiogenesis inducers.

## Results

### Inhibition of endothelial RhoA expression affects angiogenesis *in vitro*

**Figure 1:**
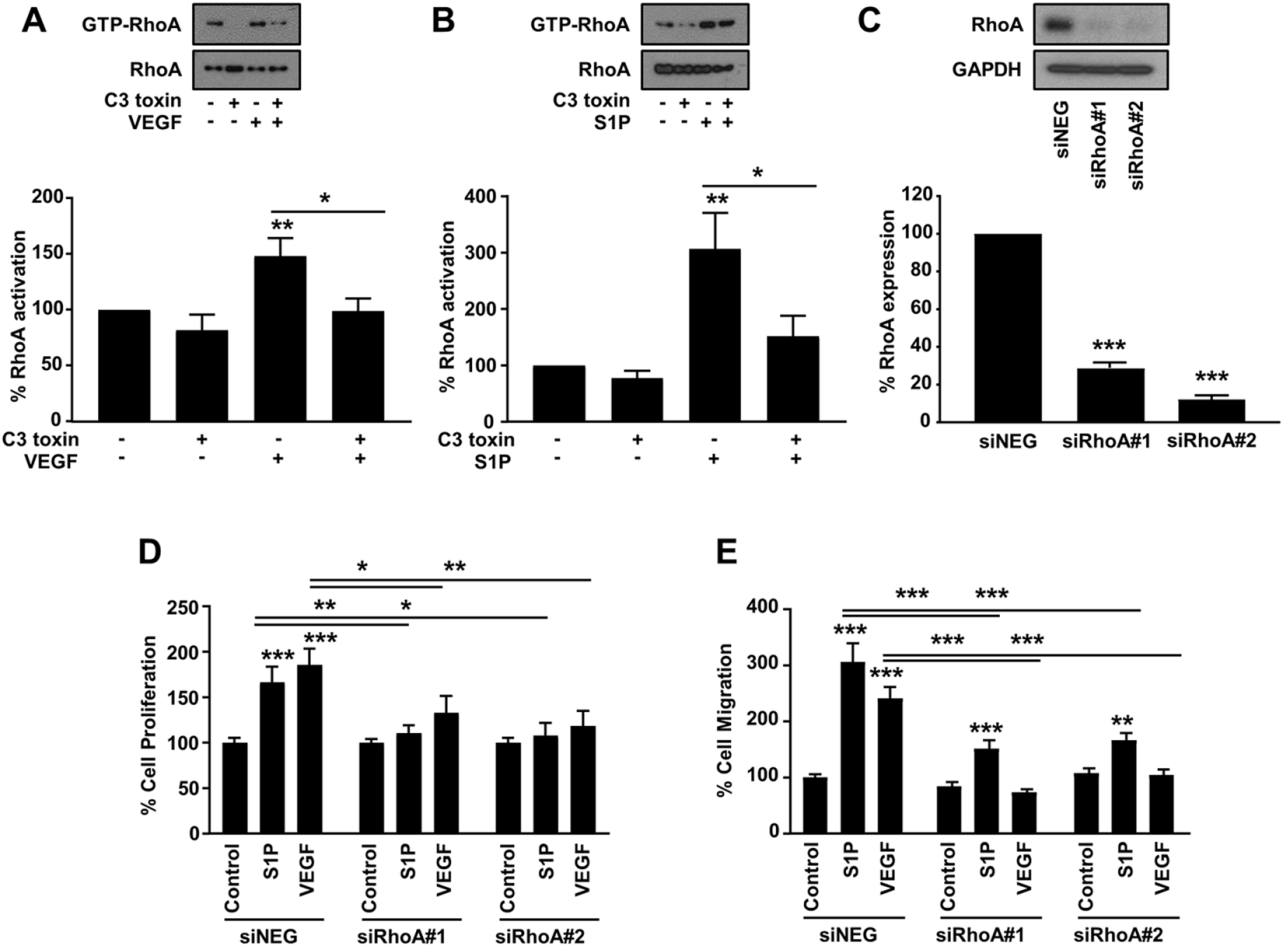
Involvement of endothelial RhoA in VEGF- and S1P-induced cell proliferation and migration. (**A,B)** Representative images (upper panel) and quantification (lower panel) of RhoA activation in HUVECs. Cells were stimulated with VEGF (100 ng/ml) (n = 5) **(A)** and S1P (500 nM) (n = 9) **(B)** in the presence or absence of C3 toxin (20 ng/ml) treatment. Full-length blots are presented in Supplementary Fig. 4. **(C)** Representative image of western blot analysis (upper panel) and quantification (lower panel) of RhoA expression after transfection with siRNA control (siNEG) and two sequences of siRNA for RhoA (siRhoA#1 and siRhoA#2) (50 nM) (n = 3). Full-length blots are presented in Supplementary Fig. 5. **(D)** Quantification of VEGF- (100 ng/ml) and S1P-induced (500 nM) cell proliferation (n = 6) and cell migration (n = 6) **(E)** of HUVECs treated with the corresponding siRNAs. *P < 0.05; **P < 0.01; ***P < 0.001.

The participation of RhoA signaling pathway downstream of VEGF-induced angiogenesis has been previously reported^10,21,22^. To investigate the role of endothelial RhoA in angiogenesis induced by stimuli activating diverse signaling pathways, we selected VEGF and S1P as representative angiogenesis inducers through tyrosine kinase receptor^23,24^ and G protein-coupled receptor signaling respectively^25^. Both VEGF and S1P stimulation induced RhoA activation in primary endothelial cells (HUVECs) and this induction was blocked by C3 toxin (exoenzyme C3) treatment (Fig. 1A,B). To identify whether RhoA inhibition affects VEGF- and S1P-induced angiogenesis *in vitro*, RhoA expression was knocked down (Fig. 1C) with siRNAs. RhoA knockdown in the endothelial cells abrogated mitogenic activity in 24 h induced by both VEGF and S1P (Fig. 1D). VEGF-induced cell migration, measured through the Boyden chamber assays, was completely abrogated by RhoA knockdown (Fig. 1E), whereas S1P-induced cell migration was partially inhibited, demonstrating that although RhoA is involved in the downstream signaling cascade, S1P but not VEGF may also elicit cell migration by additional mechanisms (Fig. 1E).

**Figure 2:**
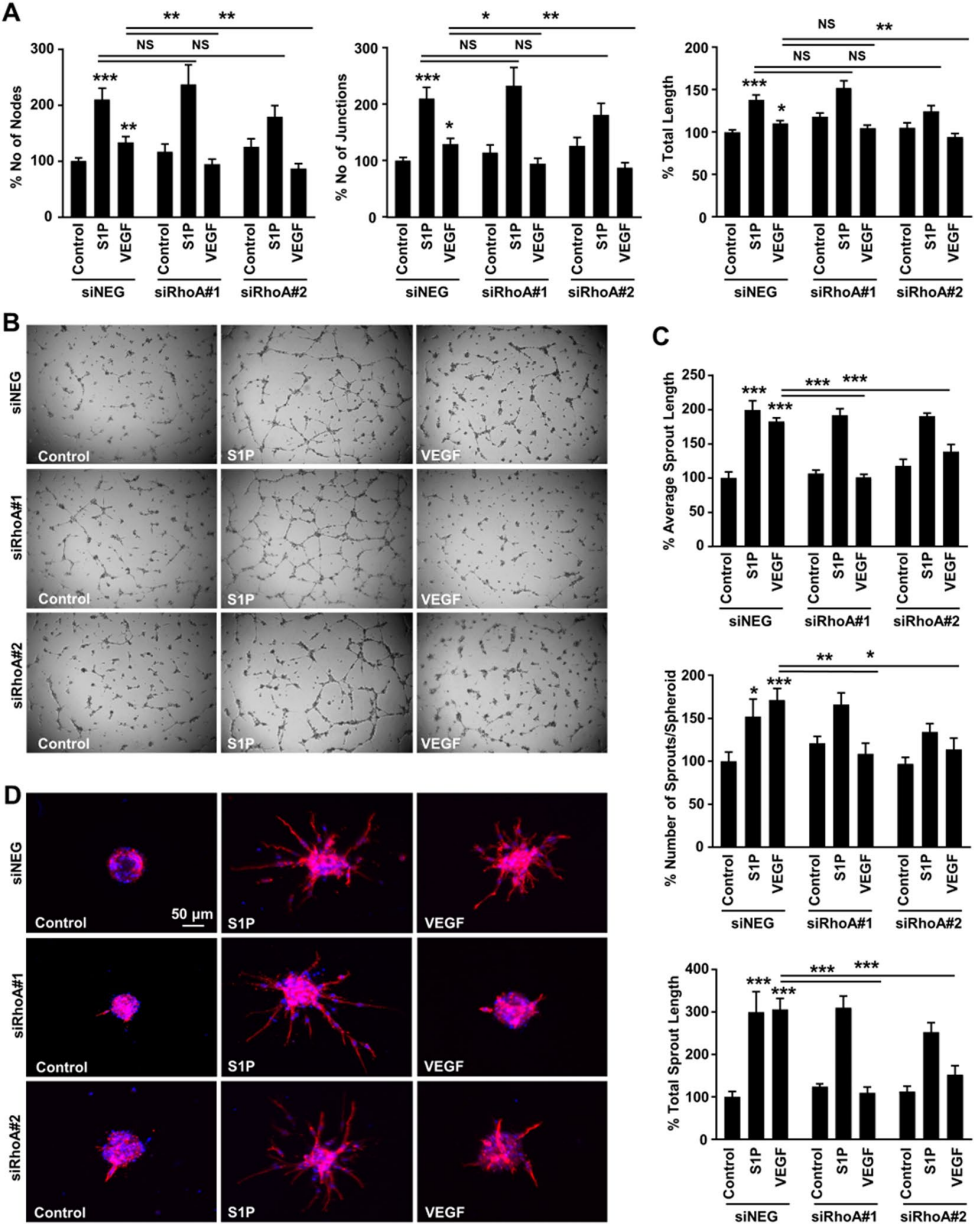
Involvement of endothelial RhoA in VEGF- and S1P-induced sprout formation *in vitro*. **(A,B)** Quantification of number of nodes, number of junctions and total junction length (n = 8) **(A)** and representative images **(B)** of the 2-D matrigel tube formation assay of siRNA-transfected HUVECs in response to VEGF and S1P stimulation. **(C,D)** Quantification of average sprout length, number of sprouts per spheroid and total sprout length (n = 3) **(C)** and representative images **(D)** of the 3-D spheroid sprouting assay of siRNA-transfected HUVECs in response to VEGF and S1P stimulation. *P < 0.05; **P < 0.01; ***P < 0.001.

The diverse significance of RhoA in VEGF- versus S1P-induced angiogenesis was more obvious in the 2-D sprouting assay (Fig. 2A,B). RhoA deficiency led to abrogation of VEGF-induced tube formation, as assessed by the number of nodes, number of junctions and total sprout length, whereas it did not affect S1P-induced tube formation (Fig. 2A,B), demonstrating that the signaling circuits governing endothelial proliferation, migration and tube formation during angiogenesis are not identical. To better clarify the role of endothelial RhoA during tube formation, a 3-D sprouting assay was introduced, where endothelial cells form spheroids and the sprouting potential is identified in a more controlled (collagen type I and methocel) environment^26,27^. In this model, RhoA knockdown abolished VEGF-induced number of sprouts, average sprout length and total sprout length, although it did not block S1P-induced sprout formation (Fig. 2C,D). On the other hand, C3 toxin treatment completely abrogated both VEGF- and S1P-induced sprouting in the same model (Suppl. Fig. 1). Since C3 toxin has been shown to ADP-ribosylate RhoA and RhoB and to a lesser extent RhoC^28^, this opens up the possibility of a compensatory mechanism of the other members of the Rho family downstream of S1P-induced sprout formation, since S1P has also been shown to activate RhoB and RhoC apart from RhoA^11^. Overall, the above data demonstrate that RhoA affects several stages of endothelial cell behavior during the angiogenic process, however its significance is variable and depends on the signaling context.

### Endothelial RhoA deficiency is not detrimental for embryonic development

**Figure 3:**
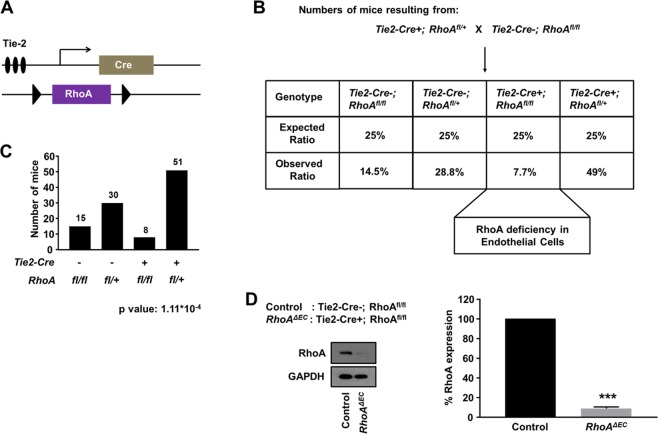
Endothelial RhoA deficiency is not detrimental for embryonic development. (**A)** Schematic diagram of RhoA transgenic mice carrying the Tie2-Cre endothelial-specific promoter and the *RhoA* floxed construct to generate RhoA-deficient endothelial cells upon endogenous promoter activation in mouse embryo. **(B)** Mating strategy to generate the *RhoA*^*f/f*^ *Tie2-Cre*+ line, with ratio of expected and obtained genotypes. **(C)** Number of mouse pups with the corresponding genotypes. **(D)** Western blot analysis of RhoA expression in isolated mouse lung endothelial cells from endothelial RhoA-deficient mice and the corresponding littermate controls (n = 3). Full-length blots are presented in Supplementary Fig. 6. ***P < 0.001.

The above data prompted us to investigate the role of endothelial RhoA during physiological angiogenesis *in vivo*. To identify whether endothelial RhoA deficiency affects vasculogenesis and angiogenesis during development, we generated mice with sustained RhoA deficiency in the endothelial cells, under the control of the Tie2 promoter. *RhoA* was deleted in endothelial cells (ECs), by crossing a conditional allele of RhoA (RhoA^f/f^) with the Tie2-Cre driver line^29^, to obtain RhoA^f/f^ *Tie2-Cre*+ mice (Fig. 3A). Tie2 is expressed in endothelial and hematopoietic cells emerging from the mesoderm^30^ and thus Tie2-promoter-driven activity is expected to occur as early as E7.5^31,32^. When the RhoA^f/+^ *Tie2-Cre*+ mice were backcrossed with the RhoA^f/f^ to obtain the final RhoA^f/f^ *Tie2-Cre*+ line, we obtained viable pups at weaning, although in a smaller than expected ratio (Fig. 3B). Even though the difference in the number of obtained versus expected RhoA^f/f^ *Tie2-Cre*+ pups was significant (Fig. 3C), the obtained RhoA^f/f^ *Tie2-Cre*+ pups did not present morphological or behavioral abnormalities from their littermate controls (RhoA^f/+^ *Tie2-Cre*+). Since the efficiency of the Tie2 promoter has been previously demonstrated^16,18,29^ and lung endothelial cells isolated from 5–6 week old mice showed almost complete RhoA deficiency in the RhoA^f/f^ *Tie2-Cre*+ mice (Fig. 3D), it is highly likely that the *RhoA* gene is efficiently deleted during embryogenesis.

### Embryos with endothelial RhoA deficiency do not present gross vascular abnormalities

**Figure 4:**
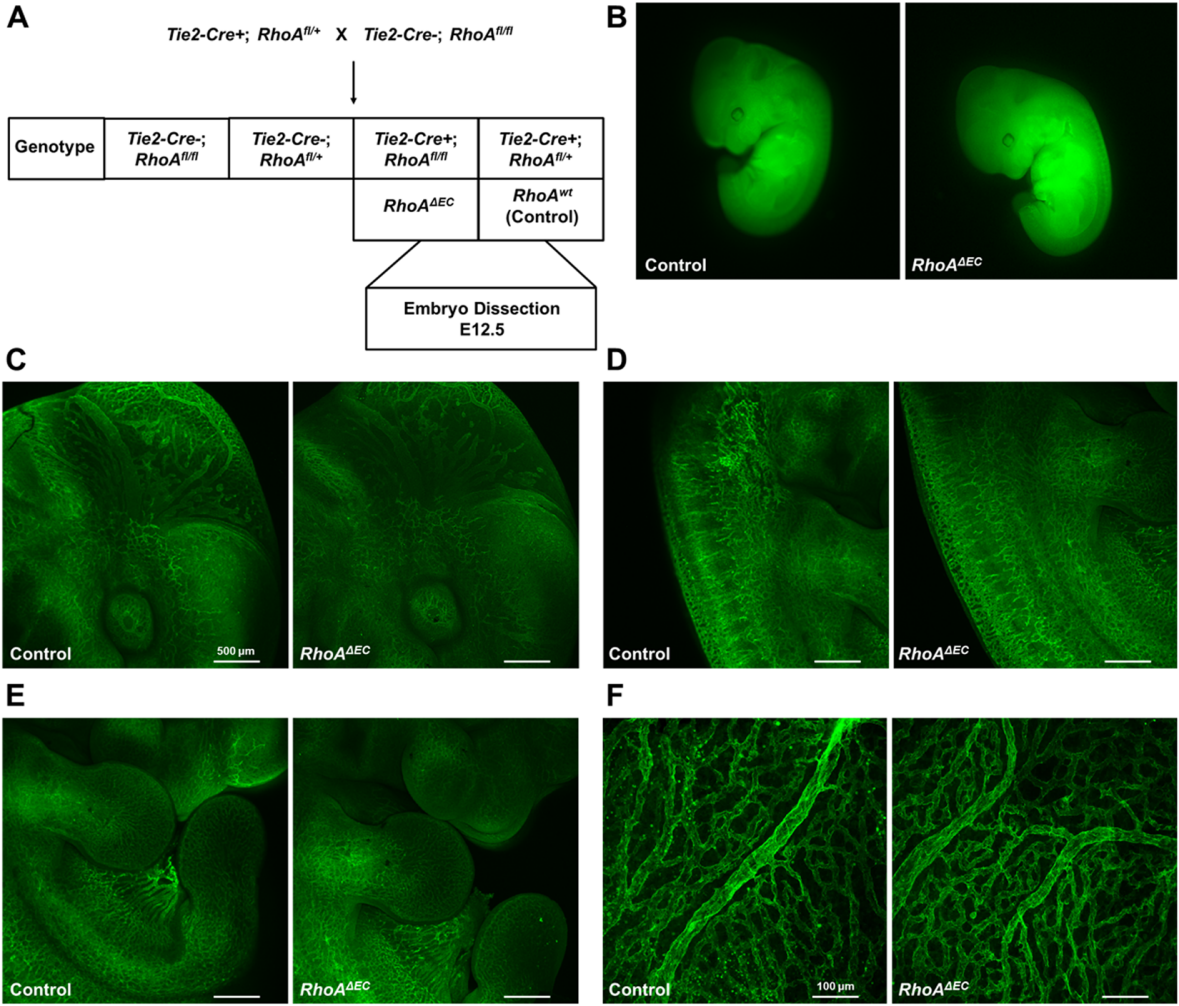
Endothelial RhoA-deficient embryos present no gross vascular abnormalities. (**A)** Schematic presentation of mating strategy for embryonic analysis experiments. **(B)** Side view of endothelial RhoA-deficient embryos and corresponding controls. **(C–F)** Whole mount CD31 staining of cranial **(C)**, trunk **(D)**, limb **(E)** and yolk sac **(F)** vessels from littermate controls (RhoA^f/+^ *Tie2-Cre*+ and endothelial RhoA-deficient (RhoA^f/f^ Tie2-Cre+) embryos (n = 4).

The decreased number of RhoA^f/f^ *Tie2-Cre*+ survivors, led us perform timed-matings to identify potential vascular deficiencies. Tie2-driven deficiency of key angiogenesis mediators is known to lead to vascular abnormalities after E10, therefore potential embryonic lethality should be visible at E12.5. To identify potential vascular deficiencies in embryos and yolk sacs, the following timed-matings were performed: RhoA^f/+^ *Tie2Cre*+ X RhoA^f/f^, the embryos were dissected at E12.5 and the RhoA^f/f^ Tie2-*Cre*+ embryos were compared with the RhoA^f/+^ *Tie2-Cre*+ littermate controls (Fig. 4A; Suppl. Fig. 2). Macroscopically, the embryos did not present gross morphological or size differences (Fig. 4B), and further vascular analysis with CD31 staining did not reveal gross vascular abnormalities in cranial vessel branching (Fig. 4C), in vessel branching in the trunk (Fig. 4D), or the limbs (Fig. 4E). Yolk sac analysis did not show gross vascular deficiencies either (Fig. 4F), which was not surprising, since no size difference in the embryos was observed.

### Breeding potential and phenotypic analysis of endothelial RhoA-deficient mice

**Figure 5:**
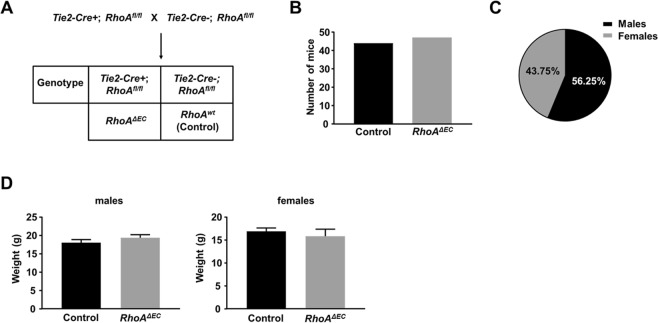
Endothelial RhoA-deficient mice present normal breeding profile and phenotypic characteristics. **(A)** Schematic representation of mating strategy for identification of breeding capability of endothelial *RhoA* knock out (RhoA^f/f^ Tie2-Cre+) versus *RhoA f/f* mice. **(B)** Number of mice with the two different genotypes. **(C)** Percentage of male versus female offspring. **(D)** Weight values between endothelial RhoA-deficient and littermate controls of male and female mice.

To identify whether the viable mice with endothelial RhoA deficiency were able to breed normally, we backcrossed the RhoA^f/f^ *Tie2-Cre*+ mice with the RhoA^f/f^ (Fig. 5A). If no deficiencies occur, 50% of the offspring should carry the Tie2 promoter, thus should be deficient for endothelial RhoA. Indeed, the offspring were tested at weaning age and almost 50% of the offspring carried the Tie2 promoter (Fig. 5B). Male versus female RhoA^f/f^ *Tie2-Cre*+ mice were obtained at normal Mendelian ratios (Fig. 5C), and mutant offspring were both males and females (not shown) denoting no sex-linked deficiencies. The endothelial RhoA-deficient mice did not present any abnormality in physical characteristics, behavior or growth rate, as also denoted from their weight measurements (Fig. 5D).

### Effect of endothelial RhoA deficiency in retinal angiogenesis

**Figure 6:**
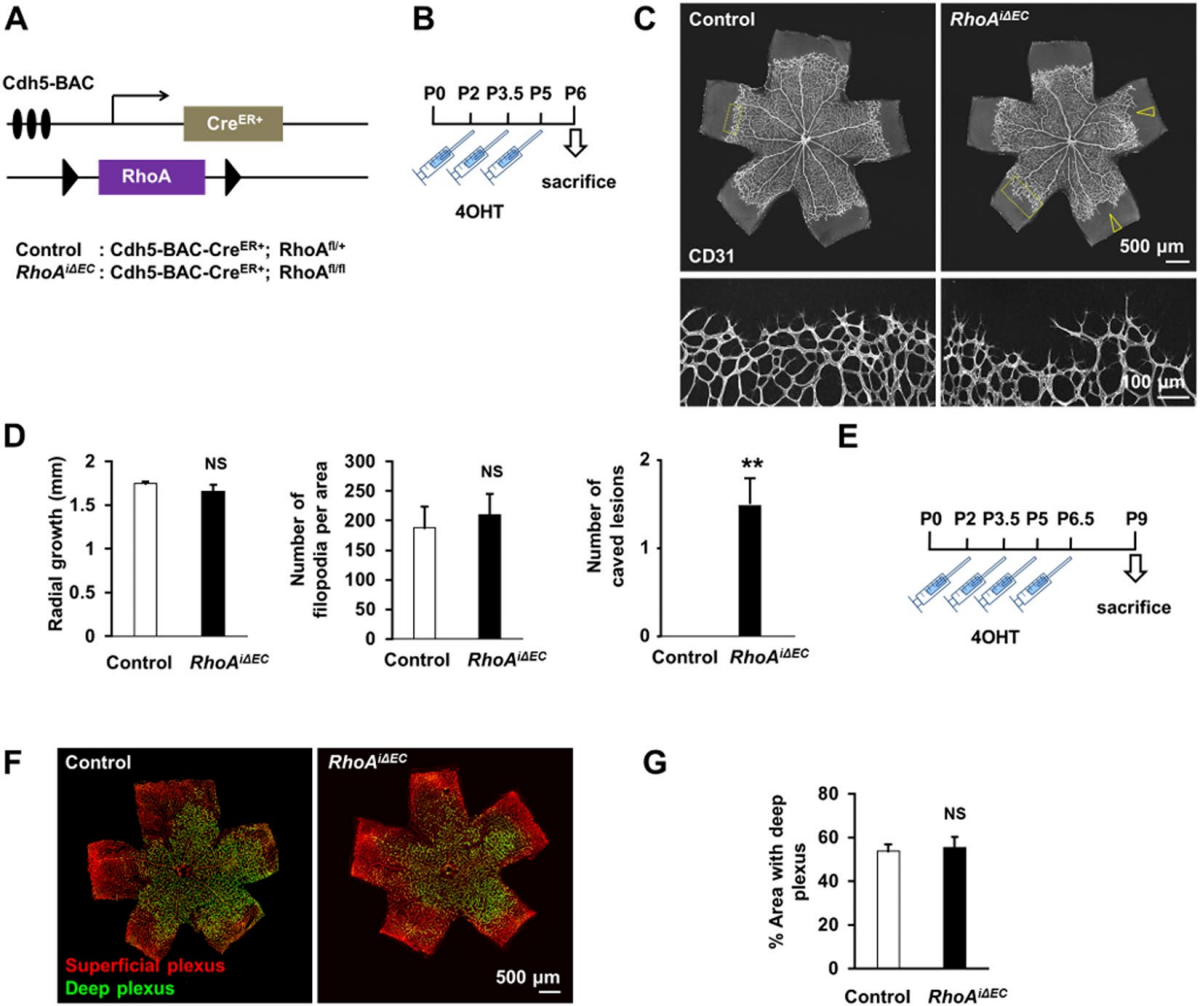
Endothelial RhoA deficiency does not affect postnatal retinal angiogenesis. (**A)** Schematic diagram of inducible endothelial-RhoA deficient mice with the Cdh5 tamoxifen-inducible promoter *Cdh5-BAC-Cre*^*ER*+^. **(B)** Experimental outline for retinal angiogenesis assay on postnatal day 6 (P6). **(C,D)** Representative images **(C)** and radial growth, number of filopodia quantifications and number of caved lesions per retina **(D)** of P6 CD31-stained retinal vessels between endothelial RhoA-deficient mice and littermate controls (n = 4). Empty arrowheads represent caved edges of the vascular fronts, observed in RhoA-deficient mice. **(E)** Experimental outline for retinal angiogenesis assay on P9. **(F,G)** Representative images **(F)** and quantification **(G)** of retinal vessels, distinguishing superficial and deep plexus stained with biotinylated isolectin B4 (IB4) (n = 3). **P < 0.01

The early postnatal mouse retina is a well-developed model for developmental angiogenesis study^33–35^. To avoid potential secondary consequences of embryonic vascular deficiencies due to *RhoA* deletion and to achieve endothelial cell-specific *RhoA* deletion at will, we used an endothelial promoter with inducible (*Cdh5-BAC-Cre*^*ER*+^), instead of sustained (*Tie2-Cre*+) activation. Therefore, we generated mice with inducible endothelial RhoA deficiency, driven by the VE-cadherin tamoxifen-inducible promoter *Cdh5-BAC-Cre*^*ER *+^^36^ (Fig. 6A). After 4-hydroxytamoxifen (4-OHT) administration during P2-P5 (Fig. 6B), as previously described^36^, we examined the developing retinas on day P6 (Fig. 6C; Suppl. Fig. 3). The pattern of retinal vascular sprouting of the endothelial RhoA-deficient retinas was mildly disrupted, resulting in an uneven growing front, characterized by ragged and caved edges. However, no significant difference was observed in the radial growth or in the number of filopodia in the retinas upon endothelial RhoA deficiency (Fig. 6D), except from the number of caved lesions per retina, which were consistently present only in the endothelial RhoA-deficient retinas (Fig. 6D). The mild, although reproducible, phenotype suggested that either RhoA in the endothelial cells is dispensable for retinal angiogenesis or that *RhoA* deletion did not result in significant reduction of RhoA protein levels at this point. To exclude the second scenario, we repeated the analysis in P9 retinas, after 4-hydroxytamoxifen (4-OHT) administration during P2-P6.5 (Fig. 6E). At P9 retinas, the remodeling of the superficial layer is almost complete, whereas the plexus formation of the deep layers is still ongoing^33^. No difference was observed in the superficial plexus (Fig. 6F), nor in the deep plexus coverage (Fig. 6F,G), demonstrating that endothelial RhoA seems to be dispensable for retinal angiogenesis.

### Study of compensatory mechanisms and effect on downstream signaling pathway

**Figure 7:**
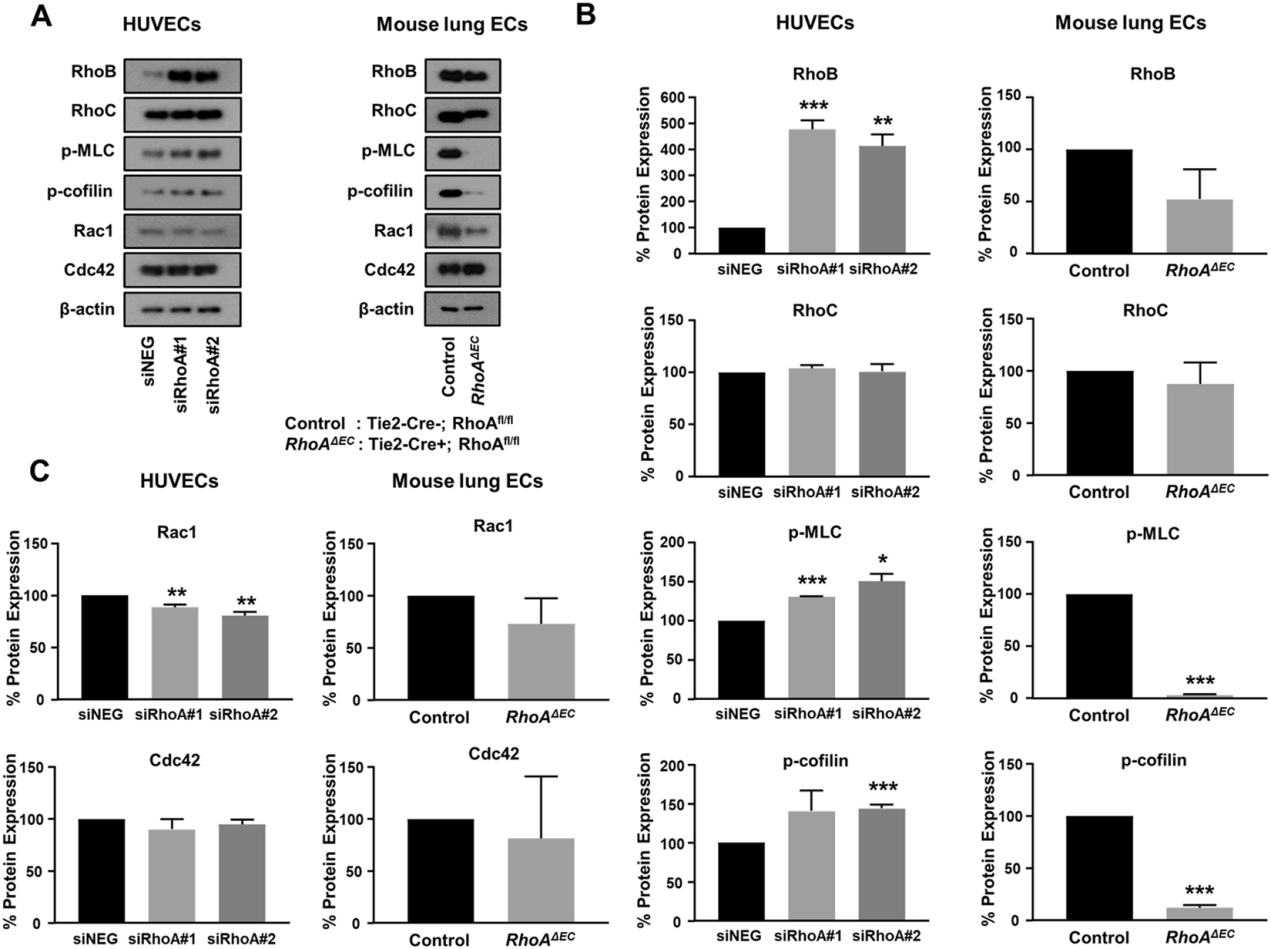
Study of compensatory mechanisms and downstream signaling pathway analysis. (**A–C)** Representative images of western blot analysis **(A)** and quantification **(B,C)** of RhoB, RhoC expression, MLC and cofilin activation **(B)** and Rac1 and Cdc42 expression **(C)** in HUVECs after transfection with siRNA control (siNEG) and two sequences of siRNA for RhoA (siRhoA#1 and siRhoA#2) (50 nM) (n = 3) and mouse lung ECs from *RhoA*^*ΔEC*^ mice and littermate controls (n = 2). Full-length blots are presented in Supplementary Fig. 7. *P < 0.05; **P < 0.01; ***P < 0.001.

Several studies have demonstrated compensatory roles of RhoB and RhoC, the other two members of the Rho GTPase family, upon RhoA deficiency^37–41^. To identify whether RhoA deficiency in human and mouse endothelial cells initiates compensatory mechanisms, we analyzed the expression of the RhoB and RhoC in HUVECs treated with siRNAs for RhoA and mouse lung endothelial cells from endothelial RhoA deficient mice and littermate controls (Fig. 7). In HUVECs, we saw that RhoA knockdown led to a significant upregulation of RhoB expression, whereas RhoC expression was not affected. We hypothesize that RhoB upregulation should compensate for RhoA deficiency, since the basal phosphorylation levels of the downstream signaling molecules, such as Myosin Light Chain 2 (MLC)^42^ and cofilin^43^ not only were not blocked, but were slightly elevated in HUVECs (Fig. 7A,B). Interestingly, in the RhoA-deficient mouse endothelial cells we did not observe RhoB or RhoC upregulation. This was verified also from the complete inhibition of the basal activation levels of MLC and cofilin upon RhoA deficiency in the murine endothelial cells (Fig. 7A,B). We further checked whether RhoA deficiency affected the basal expression levels of Rac1 and Cdc42 (Fig. 7A,C). The expression of Cdc42 was not affected upon RhoA deficiency in either human or mouse endothelial cells, whereas Rac1 levels were slightly inhibited in HUVEC and presented an inhibitory tendency in mouse endothelial cells, however that inhibition was not significant (Fig. 7A,C).

### Evaluation of RhoA-deficient mouse endothelial cells on angiogenesis *in vitro*

**Figure 8:**
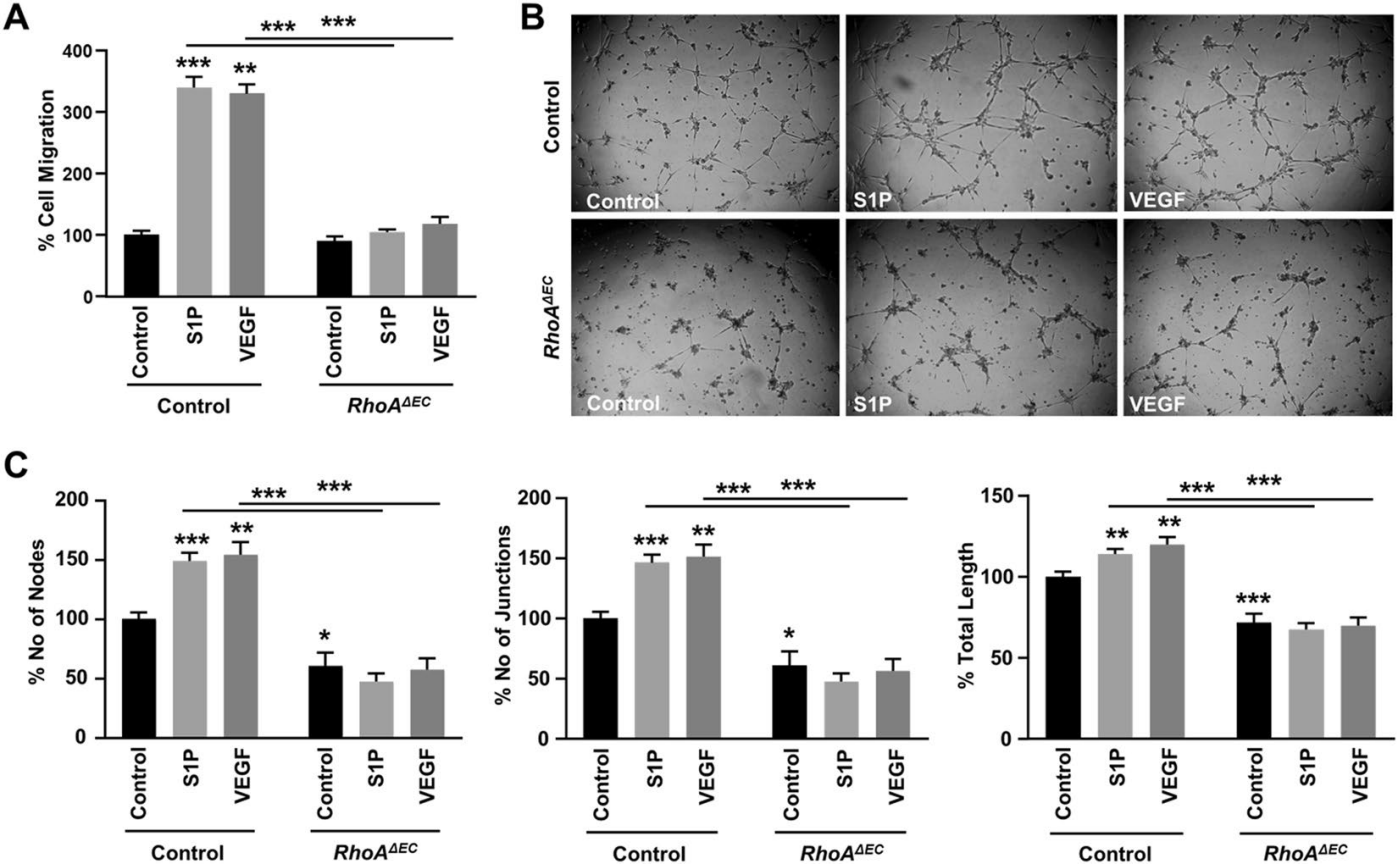
Effect of endothelial RhoA deficiency on VEGF- and S1P-induced cell migration and sprout formation *in vitro*. Quantification of mouse VEGF- (50 ng/ml) and S1P-induced (250 nM) cell migration (n = 3) **(A)** and sprout formation (n = 3) **(B,C)** of mouse lung ECs from *RhoA*^*ΔEC*^ mice and littermate controls. **(B)** Representative images and **(C)** quantification of number of nodes, number of junctions and total junction length. *P < 0.05; **P < 0.01; ***P < 0.001.

The difference in compensatory mechanisms upon RhoA deficiency in human versus mouse endothelial cells prompted us to evaluate the response of the murine endothelial cells upon S1P- and VEGF-induced angiogenesis *in vitro* (Fig. 8). RhoA deficiency completely abrogated both S1P- and VEGF-induced cell migration (Fig. 8A) and tube formation (Fig. 8B,C), assessed by the number of nodes, junctions and total sprout length. Furthermore, in the tube formation experiments (Fig. 8C) the basal angiogenesis levels upon RhoA deficiency were significantly lower, which was significant in all quantification parameters (number of nodes, junctions and total sprout length), and consistent with the reduced activation levels of the downstream signaling pathway upon RhoA deficiency in the murine endothelial cells.

## Discussion

It has been demonstrated that small GTPases regulate biological functions in endothelial cells and are considered key mediators of the angiogenic process^16–19^. In the present study we aimed to delineate the precise role of RhoA in angiogenesis *in vitro* and *in vivo*. Here we identified that RhoA is activated by both tyrosine kinase and G protein-coupled receptor ligands and regulates some of the downstream *in vitro* angiogenic functions. RhoA knockdown blocked endothelial cell proliferation induced by VEGF and S1P, as well as VEGF-induced cell migration and tube formation, whereas it partially blocked S1P-induced cell migration and did not affect S1P-induced tube formation in either 2-D or 3-D sprouting assays. Endothelial-specific *RhoA* deletion *in vivo* through the Cre-lox system was not detrimental for embryonic development. The endothelial-specific RhoA-deficient mice, although fewer than expected, did not present anatomic or behavioral deficiencies and gave rise to mutant offspring in the expected Mendelian ratios. Inducible endothelial *RhoA* deletion led to a mild phenotype, without significantly affecting retinal angiogenesis. These findings provide direct evidence that although endothelial RhoA is important for endothelial cell functions triggered by angiogenic stimuli *in vitro*, its loss can be compensated during physiological *in vivo* angiogenesis.

Our *in vitro* data on VEGF-induced RhoA activation is in accordance with previous findings^10,21,22^. RhoA knockdown in HUVECs demonstrated that RhoA blockade abrogates VEGF-induced migration, confirming previous data with dominant negative RhoA mutant (RhoA-19N) overexpression^21,44^. VEGF-induced cell migration was potently blocked with siRNA treatment, similarly to the blockade caused by RhoA-19N overexpression^21^, or by inhibition of Rho kinase or ROCK, the direct downstream RhoA target^10,22^. Similarly, RhoA knockdown in HUVECs efficiently blocked VEGF-induced tube formation. Similar findings had been previously obtained in Human Microvascular Endothelial Cells and in the MS1 endothelial-like cell line after treatment with the ROCK inhibitor Y-27632 or by knockdown experiments of Rho kinases respectively^10,22^. However, previous studies demonstrating the effect of ROCK inhibition on angiogenesis may not reflect the effect of RhoA depletion. RhoA controls other downstream pathways^45^, that may be required for angiogenesis, so it is not necessarily expected that ROCK inhibition would phenocopy RhoA inhibition *in vitro* or *in vivo*.

S1P is a bioactive lipid mediator, abundant in plasma, which participates in several physiological processes, including angiogenesis, through GPCR activation^25^. S1P binds and signals through five receptors, S1PR_1–5_, all GPCRs, from which S1PR_1–3_ are expressed in endothelial cells, with S1PR_1_ presenting higher expression levels than S1PR_2_ and S1PR_3_^46^. S1PR_1_ couples mostly with Gα_i_, S1PR_2_ with Gα_12/13_ and S1PR_3_ with Gα_q_^25^. The significant role of S1P on angiogenesis *in vivo* is evident from the fact that S1PR_1_ deficiency caused embryonic lethality due to vascular deficiencies and severe hemorrhage^47^. This phenomenon was exacerbated in the S1PR_1/2/3_ triple deficient mice^48^, whereas the single deficiency of S1PR_2_ or S1PR_3_ did not present viability issues^49,50^. Previous studies have shown that S1P induces RhoA activation in endothelial cells^11,51,52^ and this activation has been studied in the context of endothelial barrier regulation. Here, we verified the activation of RhoA downstream of S1P signaling, S1P’s stimulating effect on endothelial cell proliferation and migration, and the RhoA knockdown experiments revealed the critical role of RhoA in both biological functions. Participation of RhoA on S1P-induced angiogenesis was previously shown with exoenzyme C3 toxin treatment, where RhoA activation was found to be downstream of S1PR_1_ and S1PR_3_^53^, and our data confirmed this finding. Regarding the role of RhoA on S1P-induced sprouting, it has been reported that RhoA has no effect on endothelial 3-D sprouting^54^, which coincides with our findings in our 2-D and 3-D sprouting systems. S1P has also been reported to exert an inhibitory role on angiogenesis, which is attributed mainly to RhoC activation, since exoenzyme C3 treatment increased sprouting formation in the 3-D sprouting assay^54^. Our data, however, showed that C3 treatment abolished the S1P-induced sprouting efficiency, without inducing basal sprouting levels. This discrepancy could be attributed to the difference of C3 toxin efficiency, as higher concentration is routinely being used for our experiments, guided by the efficiency to block RhoA activation.

Global deficiency of RhoA is not compatible with life^15^. Tie2 (tunica intima endothelial kinase 2) is a tyrosine kinase receptor, expressed in endothelial and some hematopoietic cells^30,31^. Although Tie2 is not considered critical for fetal hematopoiesis^55^, Tie2-deficient mice have been reported to die at E10.5 due to vascular and hematopoietic deficiencies^30,56^. Similarly, Tie2 promoter-driven deficiency leads to embryonic lethality around E9-10.5^16,18,57^. Therefore, potential deficiencies due to Tie2-driven excision should be evident prior to E12.5, where the embryo dissection took place. We had previously demonstrated that global RhoA activation deficiency, through the combined global deficiency of two RhoGEFs, PDZ-RhoGEF and LARG, led to embryonic lethality before E10.5, due to partial branching failure of the cranial vessels and deficient embryonic vascular network in the placenta^20^. Here, however, we found that although RhoA deficiency in the endothelial cells could partially affect the viability of the mutant embryos, is not detrimental for life. A possible explanation may be that RhoA deficiency in the endothelium may not be strictly responsible for embryonic lethality due to vascular abnormalities, but RhoA expression in the surrounding tissues should play a significant role. Although the precise mechanism of RhoA functions in each cell type has not been described in detail, nor their role on paracrine signaling between different cell types, that scenario cannot be excluded, since RhoA regulates the activity of several transcription factors, such as the (SRF)/MAL, the AP-1 or the NF-kB^58^. RhoA activation in the surrounding tissues seems to play compensatory role for endothelial cell functions in the absence of endothelial RhoA, which when lost, either in the *in vivo*^20^ or *in vitro* settings^10,21,22^ (and our data), exacerbates the vascular deficiencies.

The mouse retina is an established post-embryonic angiogenesis model^59^. Retinal vasculature develops postnatally through sprouting angiogenesis^60^, following a well-defined sequence of events. From birth (P0) till post-natal day 6 (P6) the retinal vessels grow from the optic disc to the peripheral margin forming a superficial vascular plexus, and around that point they also start invading the retina to form the deeper vascular layers^33,36^. Our experiments revealed that endothelial RhoA deficiency led to mild phenotype, characterized by uneven retinal vascular front with ragged and caved edges, however the major quantitative parameters were not affected. Similar mild phenotype has been previously reported for sema3E, plexin-D1 and neuronal VEGFR2 deficiencies^36,61^, and here could suggest that RhoA is mainly dispensable during retinal angiogenesis or that RhoA gene deletion may not result in significant reduction of RhoA protein levels, although the efficiency of the inducible promoter has been previously demonstrated^36^. Our findings are generally in accordance with previous experiments with retinal explants where treatment with RhoA pathway inhibitors did not present differences in the basal levels of capillary vessel outgrowth, although exogenous VEGF-induced outgrowth was significantly inhibited^22^.

In the absence of endothelial RhoA *in vivo*, compensation from the other members of the family of Rho GTPases cannot be excluded. RhoC shares ~88% amino acid homology with RhoA, both are equally regulated by most RhoGEFs and they both regulate actin cytoskeleton^62,63^. Although they have different spatial localization patterns, suggesting distinct roles^64^, there are studies demonstrating functional redundancy. RhoA-deficient cells present no deficiencies in forming actin-rich protrusions, RhoA-deficient mouse fibroblasts present no significant actin cytoskeleton abnormalities, suggesting functional compensation from RhoC^39,40^, while similar compensatory role has also been reported in lung adenoma formation *in vivo*^41^. Our data are in line with a previous study reporting the compensatory role of RhoB upon RhoA deficiency, leading to activation of downstream targets, such as MLC and cofilin^38^. MLC and cofilin phosphorylation could also be endothelial type-specific, rather than species-specific. Although RhoA controls MLC phosphorylation in mouse lung ECs, this may not be the case for retinal mouse ECs, which could explain the lack of phenotype with RhoA depletion, and which warrants further investigation.

Both *in vivo* models included in this study are well described models for physiological angiogenesis^35^. Although physiological and pathological angiogenesis share many common characteristics, they also have significant differences^65^. Vessels driven by pathological angiogenesis, such as tumor-induced angiogenesis, are disorganized and leaky^66^. The signaling cascades in pathological angiogenesis have different origin and are more persistent, although not fully elucidated to date^67^. Moreover, the same receptors or signaling pathways may induce diverse outcome, based on the microenvironmental context. An example is the S1P_2_R pathway, which although induces pathological neovascularization, at the same time blocks hypoxia-triggered revascularization in the retina^68^. The transcriptomic profile of endothelial cells between physiological and pathological angiogenesis differs: tumor endothelial cells overexpress certain proteins, such as Doppel or Vscp, which present limited or no expression during developmental angiogenesis^69^. Some angiogenesis mediators, such as VEGF, basic Fibroblast Growth Factor (bFGF) or angiopoietins are known to mediate both physiological and pathological angiogenesis, whereas others, such as Cox2, Placental Growth Factor (Plgf), α_v_β_3_ integrin, nitric oxide and TSP-2 mediate pathological angiogenesis without affecting the developmental one^70,71^. Even in the case of VEGF, blockade of VEGF164 potently suppressed pathological neovascularization, while it had no effect on physiological neovascularization^72^, highlighting the differences in the molecular mechanisms regulating physiological versus pathological angiogenesis.

In summary, we report here that although the small GTPase RhoA regulates *in vitro* angiogenesis, its role during developmental angiogenesis may not be as significant. Additional work will be necessary to elucidate the compensatory mechanisms upon RhoA deficiency in the context of physiological versus pathological angiogenesis, which may help expose the potential of RhoA as an anti-angiogenic therapeutic target.

## Methods

### Antibodies and reagents

Cell culture reagents were purchased from Gibco^TM^ (Life technologies, Carlsbad, CA). ECGS (Cat#356006) was purchased from Corning (San Jose, CA), 5000U/ml Heparin solution was purchased from Hospira (NDC#63739-920-11; Lake Forest, IL). Exoenzyme C3 from *Clostridium botulinum* (Cat#CT03) was purchased from Cytoskeleton (Denver, CO). The following pre-designed siRNAs: Silencer® Select Negative Control #1 (Cat No: 4390846), siRhoA (Cat Nos: s758 and s759), fluorescent streptavidin conjugates (Molecular Probes), Alexa 488 fluorescence-conjugated IgGs (Molecular Probes) and the Halt Protease and Phosphatase Inhibitor Cocktail (Cat#PI78445) were purchased from Thermo Fisher Scientific (Waltham, MA). DharmaFECT 1 (Cat# T-2001-02) transfection reagent was purchased from Dharmacon (Lafayette, CO). Reduced Growth Factor (RGF)-Basement Membrane Extract was purchased from Trevigen (Gaithersburg, MD). Sphingosine-1-Phosphate (S1P) (Cat# NC9978856), rat anti-mouse CD31 primary antibody (Clone MEC13.3, Cat#553370, BD Pharmingen) and donkey anti-rat Alexa488 secondary antibody (Cat#A-21208, Life Technologies) were purchased from Fisher (Hampton, NH). Glutathione Sepharose^TM^ 4B beads (Cat#45-000-139), Collagen type I (Cat#CB354249), rat anti-mouse CD102 (ICAM2) antibody (Clone 3C4, mlC2/4 Cat#553325), 4-Hydroxytamoxifen (Cat#50-136-5306), Alexa Fluor 594 Phalloidin (Cat#A12381), Hoechst Trihydrochloride Trihydrate (Cat#33342), Triton X-100 (Cat#BP151-100), Bovine Serum Albumin (BSA), Tris-HCl, NaCl, phenylmethylsulfonyl fluoride, aprotinin, leupeptin and other chemicals were also purchased from Fisher (Hampton, NH). Rabbit anti-RhoA (Cat# 21017) was purchased from NewEast Biosciences (King of Prussia, PA). Hamster anti-CD31 antibody (2H8 clone; Cat# ENMA3105) was purchased from Chemicon (Temecula, CA). Cy3/Cy5 DyLight549/DyeLight649-conjugated IgGs were obtained from Jackson ImmunoResearch (West Grove, PA). Human (Cat# SRP3182-10UG) and mouse (Cat#V4512) VEGF, Gelatin 2% (Cat#G1393-100ML), biotinylated isolectin B4 (IB4; Cat#L2140) and Methyl Cellulose (Cat#M0512-250G), Mayer’s Hematoxylin Solution (Cat# MHS32-1L), JumpStart REDTaq ReadyMix Reaction Mix (Cat#P0982) were purchased from Sigma Aldrich (St. Louis, MO). 32% Paraformaldehyde (formaldehyde) aqueous solution (Cat#15714-S) was purchased from Electron Microscopy Systems (Hatfield, PA). Primary antibodies against RhoA (Cat#2117; 1:1000), RhoB (Cat#63876; 1:1000), RhoC (Cat#3430; 1:1000), Phospho-Myosin Light Chain (Cat#3674; 1:1000), Phospho-Cofilin (Cat#3313; 1:1000), Cdc42 (Cat#2462; 1:1000), β-actin (Cat#3700; 1:2000) and GAPDH (Cat#5174; 1:2000) were purchased from Cell Signaling Technology (Beverly, MA). Mouse anti-Rac1 primary antibody was purchased from BD Biosciences (San Jose, CA). Goat anti-rabbit (Cat#4010-05, 1:50,000) and anti-mouse (Cat#1010-05, 1:50,000) secondary antibodies were from Southern Biotech (Birmingham, AL). Immobilon Western Chemiluminescent HRP substrate (Cat# WBKLS0500) was from Millipore (Burlington, MA).

### Cell lines and culture procedures

Cells were maintained at 37 °C with 5% CO_2_ in a humidified environment, following standard protocols^73,74^. Human Umbilical Vein Endothelial Cells (HUVECs) were isolated from human umbilical cords under Institutional Review Board (IRB)-approved protocol A15-3891 (Texas Tech University Health Sciences Center Institutional Review Board) in accordance with relevant guidelines and informed consent was obtained from all donors. HUVECs were used between passages 1 and 6 and all experiments were performed in HUVECs from at least three different donors, unless stated otherwise. They were routinely cultured in M199 medium (Corning) (Cat#MT10060CV), supplemented with 15% Fetal Bovine Serum (FBS) (GIBCO^TM^) (Cat#10438026), 150 μg/ml Endothelial Cell Growth Supplement (ECGS), 5 U/ml heparin sodium and 1X Antibiotic-Antimycotic solution (GIBCO^TM^) (Cat#15240-062) (EC complete medium).

### Mice

Animal studies were carried out according to Texas Tech University Health Sciences Center (TTUHSC) Institutional Animal Care and Use Committee (IACUC)-approved protocols, in compliance with the Guide for the Care and Use of Laboratory Animals. All mice used were maintained on a C57BL/6 background and both males and females were used for experiments. The generation of the floxed alleles of the genes encoding RhoA has been described previously^40^. Endogenously-regulated endothelial-specific *RhoA* knockouts were obtained by crossing the *RhoA* floxed mice with mice carrying a Cre-mediated recombination system, driven by the Tie2 promoter (*Tie2-Cre*)^29^. Conditionally-regulated endothelial-specific *RhoA* knockouts were obtained by crossing the *RhoA* floxed mice with mice carrying a tamoxifen-inducible Cre-mediated recombination system, driven by the Cdh5 promoter (*Cdh5-BAC-Cre*^*ER+*^)^36^. 4-hydroxytamoxifen (40 μg) was subcutaneously injected at postnatal days P2, P3.5, P5 and P6.5. Schemes of the protocol for gene deletion are presented in Fig. 6B,E.

Genotyping assay for *RhoA*^*f/f*^ *Cdh5-CreERT*^2^ and *Tie2-Cre* mutants was performed by Polymerase Chain Reaction (PCR) on mouse genomic DNA extracts from tail biopsies. The JumpStart REDTaq ReadyMix Reaction Mix was used for PCR, along with the following primers: *RhoA*^*f/f*^ Forward: 5′-TCTCTGCACTGAGGGAGTTAGG-3′, *RhoA*^*f/f*^ Reverse: 5′-GTACATACAGGGAATGGAAACAAGG-3′, *Cre* Forward: 5′-GCGGTCTGGCAGTAAAAACTATC-3′, *Cre* Reverse: 5′-GTGAAACAGCATTGCTGTCACTT-3′, *Tie2-Cre* Forward: 5′-CGATGCAACGAGTGATGAGG-3′, *Tie2-Cre* Reverse: 5′-CGCATAACCAGTGAAACAGC-3′.

### Cell transfection

HUVECs were transfected with siNEG, siRhoA#1 or siRhoA#2, using DharmaFect1, following manufacturer’s instructions. Briefly, the cells were cultured in a 6-well till 80% confluency, then the cells were starved for 1 h with M199 medium without antibiotics (starvation medium). Meanwhile, 500 nM siRNA solution in 200 μl of M199 starvation medium was prepared from the stock solution and Dharmafect was also diluted 20X in M199 starvation medium in another vial to reach the volume of 200 μl. After 5 min incubation in room temperature the siRNA vial content was transferred to the Dharmafect one, the solution was briefly vortexed and incubated for 15 min more in room temperature. Then the 400 ul of the final solution was added dropwise in the 1600 ul of M199 starvation medium per well (50 nM final concentration) and the cells were incubated for 6 h. After the incubation period the medium was replaced with EC complete medium.

### Cell proliferation assay

Cell proliferation of HUVECs was evaluated through the MTT (3-[4, 5-dimethylthiazol-2-yl]-2, 5-dimethyltetrazolium bromide) colorimetric assay, as previously described^75^. HUVECs were seeded at a density of 2 × 10^4^ cells/well in gelatin-coated 24-well plates and grown in complete medium (500 μl/well) for 24 h, prior to siRNA transfection. After 32 h of incubation in EC complete media, the medium was replaced with M199 containing 0.1% BSA. After 16 h the medium was replaced again with fresh M199 containing 0.1% BSA, as well as the tested agents and the cells were further incubated for 24 h. At the end of the incubation period, 50 μl of MTT stock (5 mg/ml in PBS) was added per well and the plates were incubated at 37 °C for 2 h. The medium was removed, the cells were washed with PBS pH 7.4 and 100 μl acidified isopropanol (0.33 ml HCl in 100 ml isopropanol) was added to each well and the plate was agitated thoroughly to solubilize the dark blue formazan crystals, formed by metabolically active cells. The solution was transferred to a 96-well plate and immediately read on a microplate reader, at a wavelength of 570 nm. Results were confirmed by direct measurement of the cells using a standard hemocytometer.

### Cell migration assay

Cell migration was performed as previously described^20^, using a 48-well Boyden chamber with an 8-μm pore size polyvinyl pyrrolidone-free polycarbonate membrane (NeuroProbe) coated with collagen. Transfected HUVECs were added to the upper chamber 48 h post-transfection, and M199 with 0.1% BSA with or without human (100 ng/ml) or mouse (50 ng/ml) VEGF or S1P (250 or 500 nM) was added to the lower chamber. After incubation for 6 h at 37 °C, the cells on the upper surface of the membrane were removed, and the cells at the lower surface were fixed with methanol and stained with hematoxylin. The cells were manually counted using a bright-field microscope (Microscoptics, IV-900).

### Tube formation assay

Matrigel tube formation assay was performed with transfected HUVECs 48 h post-transfection, as previously described^74,76^. Briefly, wells of a 96-well culture plate were coated with 40 μl/well RGF-Basement Membrane Extract (Trevigen, Cat #3433) and were left to polymerize for 20 min at 37 °C. After polymerization, 10^4^ cells suspended in 100 μl of M199 0.1% BSA were added to the respective wells. Human (100 ng/ml) or mouse (50 ng/ml) VEGF and S1P (250 or 500 nM) were added in the medium and after 6 h incubation at 37 °C, the medium was removed, the cells were fixed, and pictures of the wells were captured using a bright-field microscope (Microscoptics, IV-900) connected with a digital camera (AmScope FMA050) at 4X magnification and later analyzed for number of nodes, number of junctions and total sprout length using the “Angiogenesis analyzer” plug-in^77^ in ImageJ software (National Institutes of Health).

### Immunoblot analysis

The immunoblot analysis was performed as described previously^73^. The cells were lysed on ice in RIPA buffer (10 mmol/L Tris-HCl, 1 mmol/L EDTA, 0.5 mmol/L EGTA, 1% Triton X-100, 0.1% sodium deoxycholate, 0.1% SDS and 140 mmol/L NaCl), supplemented with protease and phosphatase inhibitors (Halt Protease and Phosphatase Inhibitor Cocktail; Thermo Scientific). Cell lysates were centrifuged at 13,000 rpm for 10 min at 4 °C and each supernatant was mixed with the appropriate volume of 5x SDS loading buffer, heated to 95–100 °C for 5 min and briefly centrifuged. Equal amounts of proteins were subjected to SDS-PAGE and transferred onto an Immobilon P, polyvinylidene difluoride membrane (Millipore, Billerica, MA). The membranes were then incubated with the appropriate primary antibodies: RhoA (1:1000), p-cofilin (1:1000), p-MLC (1:1000), RhoB (1:1000), RhoC (1:1000), Cdc42 (1:1000), β-actin (1:2000) GAPDH (1:2000) (all from Cell Signaling Technology, Beverly, MA), or Rac1 (1:1000) (from BD Biosciences). As a secondary antibody, goat anti-rabbit was used (1:50000). The antigens were visualized using the Immobilon Western Chemiluminescent HRP substrate (Millipore), according to manufacturer’s instructions. The protein levels that corresponded to immunoreactive bands were quantified using the Image PC image analysis software (Scion Corp., Frederick, MD) and ImageJ image analysis software (National Institutes of Health).

### Rho GTPase pull-down assay

Rho activation in cultured cells was assessed as follows^20,42^: after serum starvation for 3 h, the cells were treated as indicated and lysed on ice in lysis buffer, containing 20 mM Hepes, pH 7.4, 0.1 M NaCl, 1% Triton X-100, 10 mM EGTA, 40 mM b-glycerophosphate, 20 mM MgCl_2_, 1 mM Na_3_VO_4_, 1 mM dithiothreitol, 10 μg/ml aprotinin, 10 μg/ml leupeptin and 1 mM phenylmethylsulfonyl fluoride. The lysates were incubated with the glutathione S-transferase-rhotekin-Rho-binding domain previously bound to glutathione-Sepharose beads (Amersham Biosciences) and washed three times with lysis buffer. Associated GTP-bound forms of Rho were released with SDS–polyacrylamide gel electrophoresis loading buffer and analyzed by western blot analysis using a monoclonal antibody against RhoA, as described above.

### Endothelial spheroid sprouting assay

The sprouting assay was performed as previously described^26,78^. Methocel preparation: Methyl Cellulose (Cat#M0512-250G, Sigma Aldrich) was autoclaved and gradually dissolved in M199 (6 g in 500 ml) under stirring at 4 °C overnight. The supernatant from a 3 h centrifugation at 3,500 g was collected and termed methocel from now on^26^. Sprouting assay: HUVECs with or without siRNA transfection (above) were resuspended in EC complete medium (described above), containing 20% methocel (Sigma) or alternatively in the presence or absence of 20 ng/ml C3 toxin. 25 μl cell suspension drops were pipetted on non-adherent plastic plates and then the plates were turned upside-down to form hanging drops in which HUVEC spheroids were formed. The plates were incubated for 24 h at 37 °C and spheroids were harvested by washing the plates with 10% FBS/PBS. Next, spheroids were centrifuged at 200 g for 5 min and resuspended in 20% FCS and 80% methocel. The collagen matrix was prepared on ice using Collagen type I (Cat#CB354249, Fisher), Medium 199 and NaOH (1 M) in 8:1:1 ratio. Additionally, 1X HEPES buffer was added to the mix for pH adjustment. The Collagen solution and the spheroid solution were mixed in 1:1 ratio and transferred to a 24-well plate. For polymerization, gels were incubated for 30 min at 37 °C. Stimulation took place with VEGF (100 ng/ml) and S1P (500 nM) in M199 (starvation medium). Pictures of the spheroids were obtained with a bright-field microscope (Microscoptics, IV-900) connected with a digital camera (AmScope FMA050) at 4X magnification and number of sprouts and sprout length were quantified with the ImageJ software (National Institutes of Health).

### Spheroid immunostaining

To obtain immunofluorescence images, the spheroids were stained for actin with phalloidin staining, according to the manufacturer’s instructions. Briefly, the spheroids were fixed by addition of 4% PFA overnight in 4 °C. After two 5-min washes with PBS, the wells were incubated with 0.2% Triton X-100 in PBS for 10 min at room temperature, followed by two 5-min washes with PBS. Then the spheroids were blocked with 3% BSA in PBS for 1 h at room temperature and incubated with Alexa 594 Phalloidin 1:100 in PBS with 3% BSA overnight at 4 °C. The extra Phalloidin was washed out with two 10-min washes with PBS and the spheroids were incubated with Hoechst 1:2000 in PBS for 10 min at room temperature. After two more 10-min washes with PBS, PBS was added in each well and the 24-well plates were covered in aluminum foil and stored at 4 °C till pictures were obtained through confocal microscopy (see respective paragraph).

### Lung endothelial cell isolation

Isolation of mouse lung microvascular endothelial cells was performed as previously described^42^. Lung endothelial cell isolation took place after weaning. Lungs were removed from two or more mice, washed in 10% FBS-DMEM, minced into 1–2 mm^2^ pieces and digested with Collagenase Type I (2 mg/ml, Cat#17-100-017, Fisher) at 37 °C for 2 h with occasional agitation. The cellular digest was filtered through a 70 μm cell strainer, centrifuged at 1,500 rpm and the cells were plated (day 0) on gelatin-coated dishes, containing endothelial cell full media (see above). On day 1, floating cells (including red blood cells) were removed and washed with PBS and fresh culture medium was added. Sheep anti-rat IgG Dynabeads (Invitrogen) were incubated with a rat anti-mouse ICAM-2 mAb (3C4) (22.5 μl Ab per 150 μl Dynabead solution) at 4 °C overnight and washed three times with PBS supplemented with 0.1% BSA and 2 mM EDTA. First purification using the pre-coated beads was done on day 5. Cells were incubated for 10 min with ICAM-2-coated beads at room temperature, under continuous agitation. This was followed by two washes with PBS and the cells were trypsinized. After trypsinization, the bead-bound cells were recovered with a magnet, washed four times, resuspended in full growth medium and plated on fresh gelatin-coated dishes. On day 10, the cells were subjected to a second purification, following the same procedure. Purity (>85%) of endothelial cells was verified by PECAM-1 staining in FACS analysis.

### Preparation of whole-mount retinas

Enucleated eyes were fixed for 20 min in 4% paraformaldehyde (PFA) in phosphate-buffered saline (PBS) and then dissected. Retinal cups were post-fixed for 30 min and then stained as described below.

### Retinal immunostaining and *in situ* hybridization

Immunohistochemistry (IHC) of whole-mount samples or tissue sections was performed as previously described^79^. At P6, the primary monoclonal antibodies used were anti-RhoA (21017; NewEast Biosciences, King of Prussia, PA), hamster anti-CD31 (2H8; Chemicon, Temecula, CA), Secondary antibodies used were Alexa 488 fluorescence-conjugated IgGs (Molecular Probes) or Cy3/Cy5 DyLight549/DyeLight649-conjugated IgGs (Jackson ImmunoResearch, West Grove, PA). At P9, blood vessels were visualized using biotinylated isolectin B4 (IB4) (Sigma), followed by fluorescent streptavidin conjugates (Molecular Probes).

### Whole mount embryo immunohistochemistry

Embryos were collected at embryonic day (E) 11.5, as previously reported^20^, with some modifications^80^. Noon of the plug day was E0.5. Embryos were dissected from their deciduas and were fixed in 4% paraformaldehyde/PBS at 4 °C overnight. They were rinsed twice with PBS, 5 min each time, and were dehydrated through immersion in the following solutions: 25% Methanol in PBS with 0.5% Triton X-100, 50% Methanol in PBS with 0.5% Triton X-100, 75% Methanol in PBS with 0.5% Triton X-100 and 100% Methanol twice, each step for 5 min with gentle shaking, at room temperature. Embryos were bleached with 5% hydrogen peroxide in methanol for 4 h at room temperature and rehydrated through immersion in the following solutions: 100% Methanol twice, 75% Methanol in PBS with 0.5% Triton X-100, 50% Methanol in PBS with 0.5% Triton X-100, 25% Methanol in PBS with 0.5% Triton X-100, and three times in PBS with 0.5% Triton X-100, each step for 5 min with gentle shaking at room temperature. Embryos were blocked in PBS with 0.1% Triton X-100 and 3% instant skim milk (blocking solution) for 2 hours and incubated with the rat anti-mouse CD31 primary antibody (Clone MEC13.3, Cat#553370, BD Pharmingen) in 1:100 dilution in the blocking solution in 4 °C for 2 days with gentle shaking. Embryos were then washed in blocking solution for 5 times, 1 h each with gentle shaking and were incubated with donkey anti-rat Alexa 488 secondary antibody (Cat#A-21208, Life Technologies) in the blocking solution in 4 °C overnight. After 5 consecutive washes of 1 h each at 4 °C, each embryo was transferred in PBS-containing wells and was imaged by confocal microscopy (below).

### Confocal microscopy

#### For spheroids

Fluorescent Images were obtained using a multiphoton microscope (A1R; Nikon, NY, USA) in the confocal mode, using a 10x objective. Each spheroid image was the projection of merging of 5–10 images of different Z focus covering an approximate 10 μm Z distance.

#### For embryos

Fluorescent Images were obtained using a multiphoton microscope (A1R; Nikon, NY, USA) in the confocal mode. Each image analyzing the vasculature of embryonic parts was the projection of merging of 800 images of different Z focus covering an 800 μm Z distance. For embryo size comparison, a fluorescent dissecting microscope (MVX10; Olympus, Pennsylvania, USA) was used.

#### For retinas

Fluorescent Images were obtained using a confocal laser scanning microscope (FV1000; Olympus, Tokyo, Japan). Quantification of cells or substances of interest was conducted on eight 500 μm x 500 μm fields of view per sample in scanned images, and numbers obtained from each of the eight fields were averages using an FV10-ASW Viewer (Olympus).

### Statistical analysis

All experiments were repeated at least three times with similar results. Statistical analysis for embryo viability (difference from expected ratio) was performed with Chi Square test, whereas for the rest of the experiments statistical analysis was performed by an unpaired two-tailed Student’s *t*-test. Data analysis was performed using GraphPad Prism version 7.00 for Windows (GraphPad Software, San Diego, CA). The asterisks in the figures denote statistical significance (NS: not significant, *P < 0.05; **P < 0.01; ***P < 0.001).

### Ethical approval and informed consent

All experimental protocols were approved by the TTUHSC IACUC committee and the experiments were performed in compliance with the Guide for the Care and Use of Laboratory Animals. For HUVEC isolation, informed consent was obtained from all participants.

## Supplementary information

Supplementary Info

## Data Availability

All data generated or analyzed in this study are included in the article (and the Supplementary Information Files).
