# Supplementary material for: Endothelial RhoA GTPase is essential for in vitro endothelial functions but dispensable for physiological in vivo angiogenesis
Source: Sci Rep. 2019 Aug 12;9:11666. doi: 10.1038/s41598-019-48053-z (PMC6690958; doi:10.1038/s41598-019-48053-z)
Supplement: Supplementary file 1 — Supplementary Info [file 41598_2019_48053_MOESM1_ESM.docx]

**Endothelial RhoA GTPase is essential for *in vitro* endothelial functions but dispensable for physiological *in vivo* angiogenesis**

**SUPPLEMENTARY INFORMATION**

Fatema Tuz Zahra^1^, Md Sanaullah Sajib^1^, Yusuke Ichiyama^2,3^, Racheal Grace Akwii^1^, Paul E. Tullar^4^, Christopher Cobos^1^, Shelby A. Minchew^1^, Colleen L. Doçi^5^, Yi Zheng^6^, Yoshiaki Kubota^2^, J. Silvio Gutkind^7^, Constantinos M. Mikelis^1,*^

1. Department of Pharmaceutical Sciences, School of Pharmacy, Texas Tech University Health Sciences Center, Amarillo, Texas, 79106, USA.
2. Department of Anatomy, Keio University School of Medicine, 35 Shinanomachi, Shinjuku-ku, Tokyo 160-8582, Japan.
3. Department of Ophthalmology, Shiga University of Medical Science, Seta Tsukinowa-cho, Otsu, Shiga 520-2192, Japan.
4. Department of Obstetrics and Gynecology, School of Medicine, Texas Tech University Health Sciences Center, Amarillo, Texas, 79106, USA.
5. College of Arts and Sciences, Marian University Indianapolis, Indianapolis, Indiana, 46222, USA.
6. Cancer and Blood Diseases Institute, Cincinnati Children’s Hospital Medical Center, University of Cincinnati College of Medicine, Cincinnati, Ohio 45229, USA.
7. Department of Pharmacology, UCSD, San Diego, California, 92093, USA.

*Correspondence:

- Constantinos M. Mikelis, Ph.D., Department of Pharmaceutical Sciences, School of Pharmacy, Texas Tech University Health Sciences Center, 1406 S. Coulter St., Amarillo, Texas, 79106, USA. Telephone: +1 806 414 9242, Fax: +1 806 356 4770, Email: constantinos.mikelis@ttuhsc.edu

| 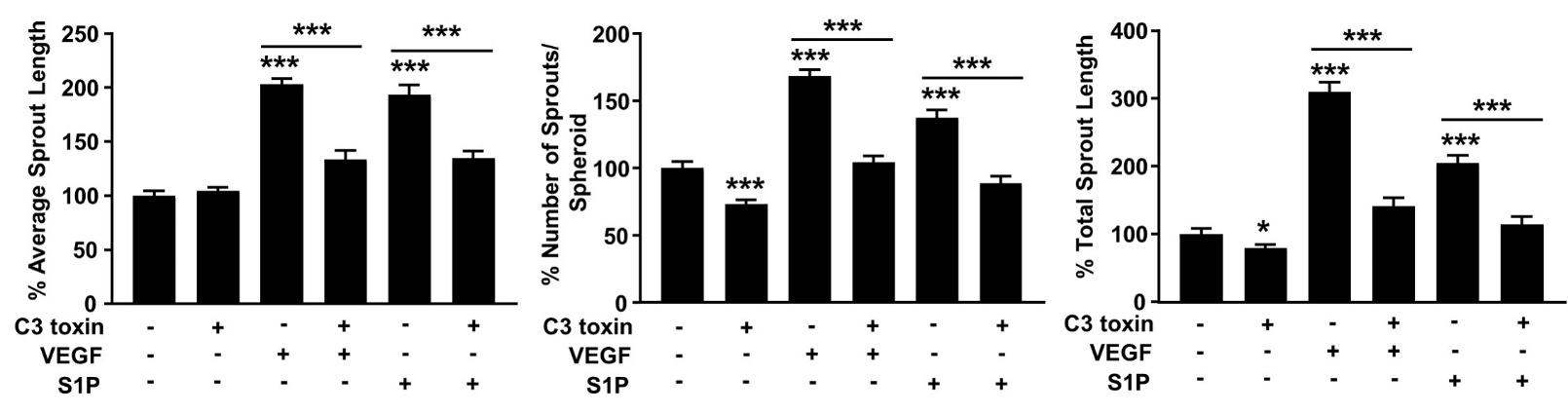 |
| --- |
| **Supplementary Figure 1. Effect of C3 toxin treatment on VEGF- and S1P-induced sprout formation *in vitro*.** Quantification of average sprout length, number of sprouts per spheroid and total sprout length in the 3-D spheroid sprouting assay of C3 toxin-treated HUVECs in response to VEGF and S1P stimulation (n=3). *P < 0.05; ***P < 0.001. |

| 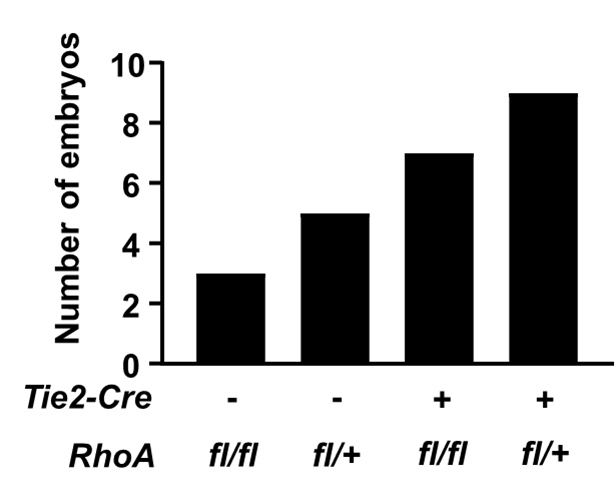 |
| --- |
| **Supplementary Figure 2. Endothelial RhoA-deficient embryos present no vascular abnormalities.** Embryos were dissected on E12.5. The number of embryos with each of the corresponding genotypes is presented. |

| 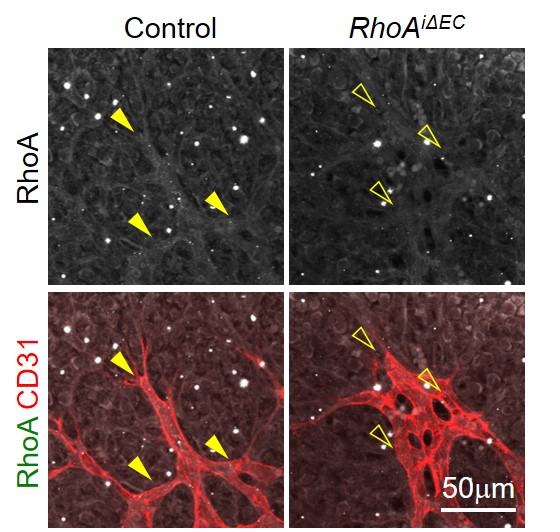 |
| --- |
| **Supplementary Figure 3. Evaluation of endothelial RhoA deficiency in P6 retinas of RhoA-deficient mice (*RhoA^iΔEC^*) and littermate controls (Control).** Representative images from CD31- and RhoA-stained retinas of RhoA-deficient mice (*RhoA^iΔEC^*) and littermate controls (Control). Arrowheads represent retinal endothelial cells stained for CD31 (lower panel) and RhoA (upper panel). |
| 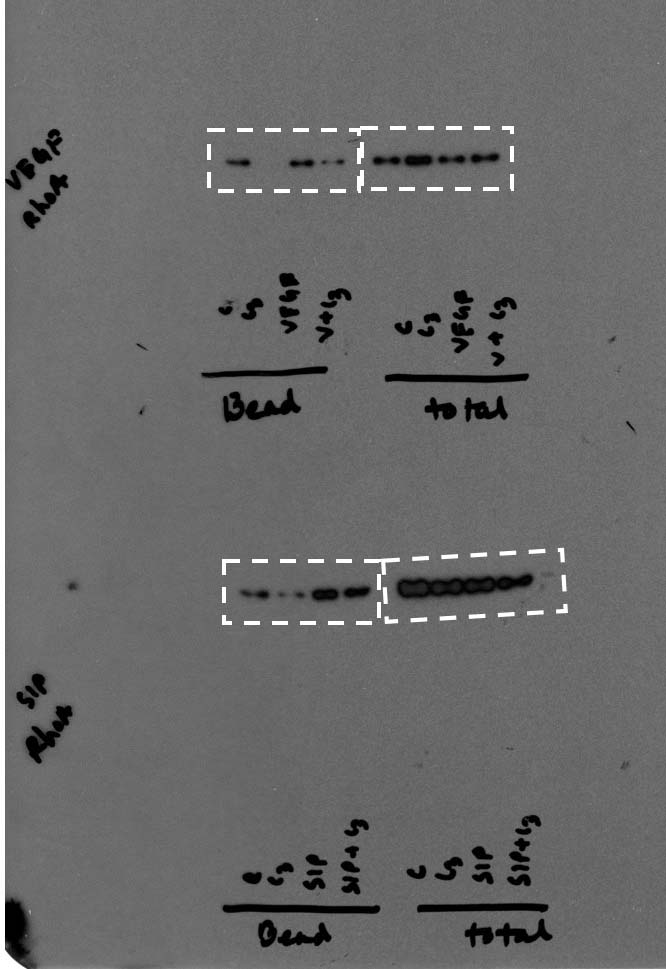 |
| **Supplementary Figure 4.** Full-length (uncut) blot of Figures 1A and 1B. Framed areas (white dashed line) correspond to the presented images in Figures 1A and 1B. |

| 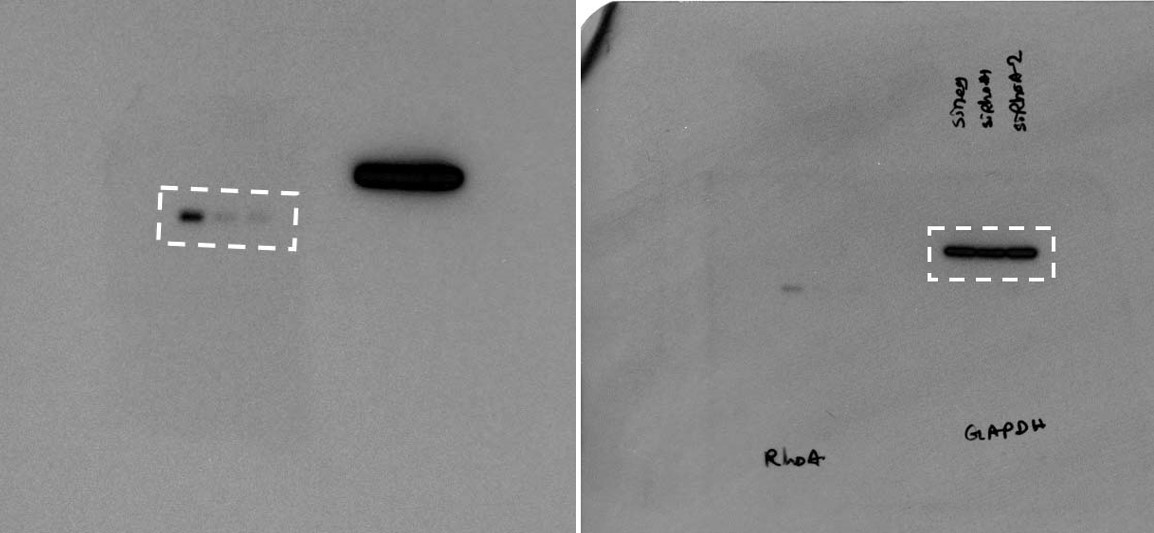 |
| --- |
| **Supplementary Figure 5.** Full-length (uncut) blots of Figure 1C. Framed areas (white dashed line) correspond to the presented images in Figure 1C. |

| 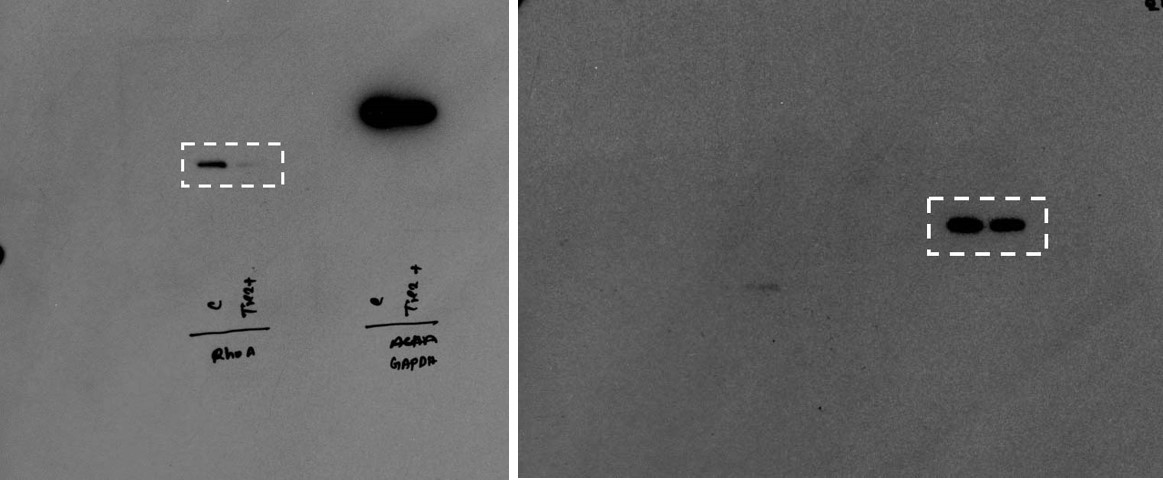 |
| --- |
| **Supplementary Figure 6.** Full-length (uncut) blot of Figure 3D. Framed areas (white dashed line) correspond to the presented images in Figure 3D. |


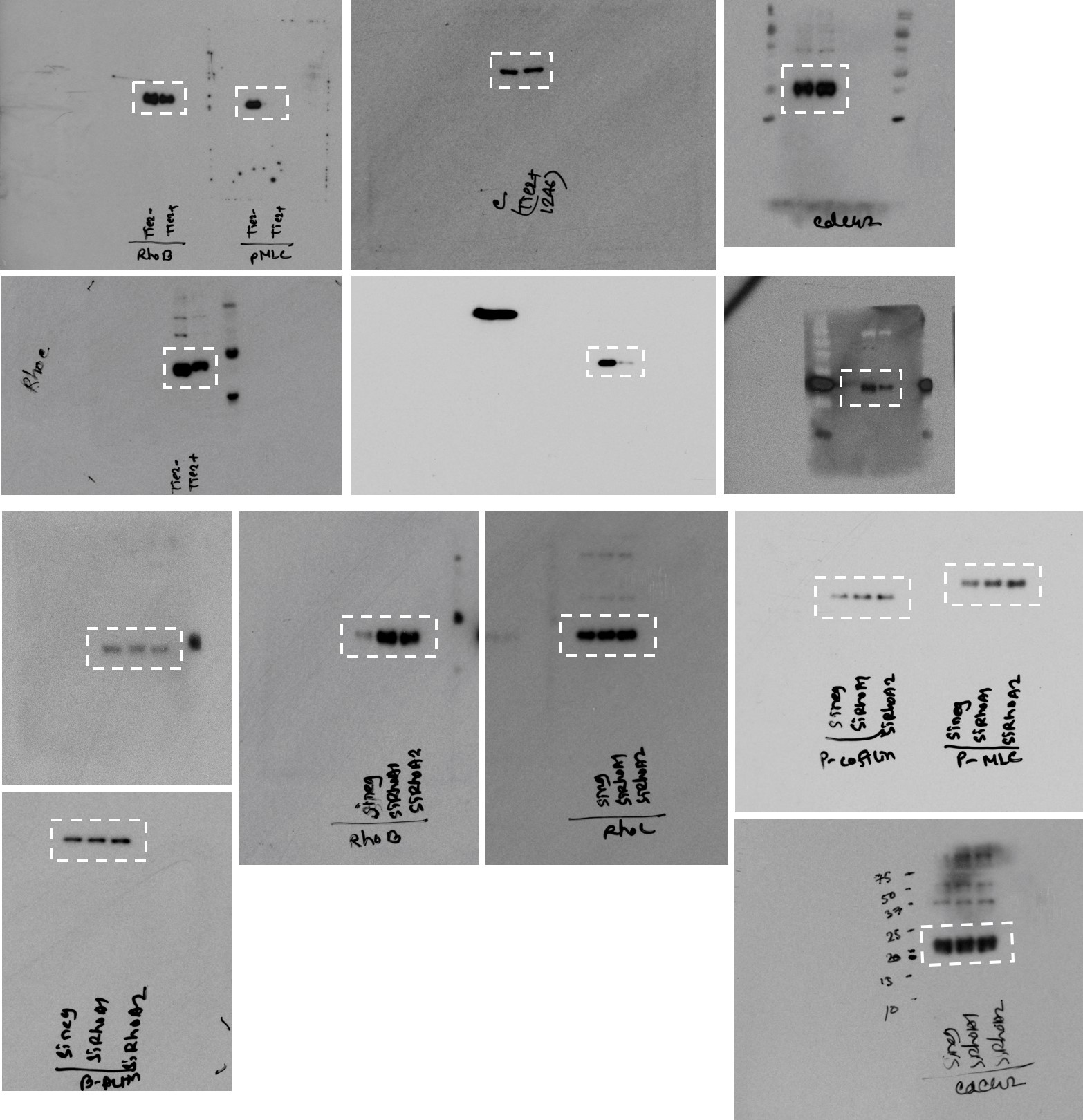


**Supplementary Figure 7.** Full-length (uncut) blots of Figure 7. Framed areas (white dashed line) correspond to the presented images in Figure 7A.
